# Supplementary material for: Interplay of Cellular Nrf2/NF-κB Signalling after Plasma Stimulation of Malignant vs. Non-Malignant Dermal Cells
Source: Int J Mol Sci. 2024 Oct 11;25(20):10967. doi: 10.3390/ijms252010967 (PMC11507371; doi:10.3390/ijms252010967)
Supplement: Supplementary file 1 [file ijms-25-10967-s001.zip › ijms-3235968-supplementary.pdf]

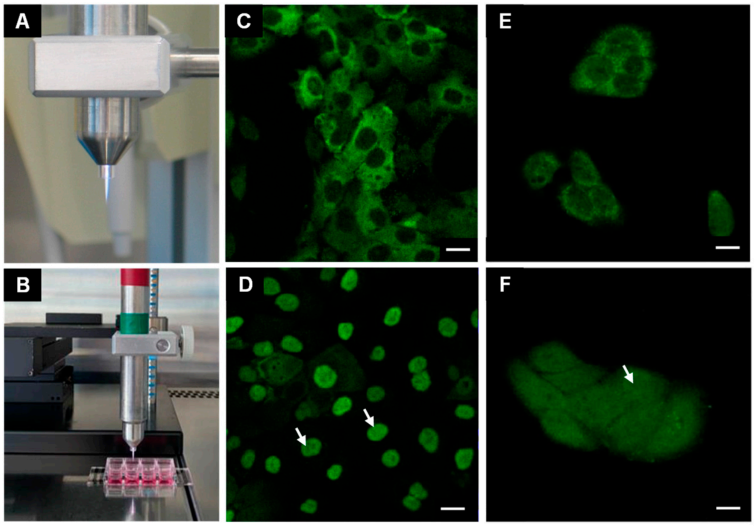

Figure S1. (**A**)Experimental setup and exemplary fluorescence images. Plasma treatment was performed using the Plasma Jet kINPen. (**B**) Setup during treatment of cells in chamberslides. (**C**) Immunofluorescence images of NF-κB in unstimulated and (**D**) Poly(I:C) treated HaCaT cells. (**E**) Nrf2 staining of unstimulated and (**F**) hydrogen peroxide stimulated HaCaT cells. For quantification, nuclei and cytosol were stained as well. Note the increased nuclear localization (arrow) in the stimulated cells (bar = 20 μm).
